# Another “String to the Bow” of PJ34, a Potent Poly(ADP-Ribose)Polymerase Inhibitor: An Antiplatelet Effect through P2Y_12_ Antagonism?

**DOI:** 10.1371/journal.pone.0110776

**Published:** 2014-10-20

**Authors:** Marie Lechaftois, Elise Dreano, Bruno Palmier, Isabelle Margaill, Catherine Marchand-Leroux, Christilla Bachelot-Loza, Dominique Lerouet

**Affiliations:** 1 EA4475-“Pharmacologie de la Circulation Cérébrale”, Faculté des Sciences Pharmaceutiques et Biologiques, Université Paris Descartes, Comue Sorbonne Paris Cité, Paris, France; 2 Inserm UMR S1140, Paris, France; 3 Faculté des Sciences Pharmaceutiques et Biologiques, Université Paris Descartes, Comue Sorbonne Paris Cité, Paris, France; University of Pecs Medical School, Hungary

## Abstract

**Background:**

Neuro- and vasoprotective effects of poly(ADP-ribose)polymerase (PARP) inhibition have been largely documented in models of cerebral ischemia, particularly with the potent PARP inhibitor PJ34. Furthermore, after ischemic stroke, physicians are faced with incomplete tissue reperfusion and reocclusion, in which platelet activation/aggregation plays a key role. Data suggest that certain PARP inhibitors could act as antiplatelet agents. In that context, the present *in vitro* study investigated on human blood the potential antiplatelet effect of PJ34 and two structurally different PARP inhibitors, DPQ and INO-1001.

**Methods and results:**

ADP concentrations were chosen to induce a biphasic aggregation curve resulting from the successive activation of both its receptors P2Y_1_ and P2Y_12_. In these experimental conditions, PJ34 inhibited the second phase of aggregation; this effect was reduced by incremental ADP concentrations. In addition, in line with a P2Y_12_ pathway inhibitory effect, PJ34 inhibited the dephosphorylation of the vasodilator stimulated phosphoprotein (VASP) in a concentration-dependent manner. Besides, PJ34 had no effect on platelet aggregation induced by collagen or PAR1 activating peptide, used at concentrations inducing a strong activation independent on secreted ADP. By contrast, DPQ and INO-1001 were devoid of any effect whatever the platelet agonist used.

**Conclusions:**

We showed that, in addition to its already demonstrated beneficial effects in *in vivo* models of cerebral ischemia, the potent PARP inhibitor PJ34 exerts *in vitro* an antiplatelet effect. Moreover, this is the first study to report that PJ34 could act *via* a competitive P2Y_12_ antagonism. Thus, this antiplatelet effect could improve post-stroke reperfusion and/or prevent reocclusion, which reinforces the interest of this drug for stroke treatment.

## Introduction

Platelet adhesion, activation and aggregation are crucial in arterial thrombosis, and therefore, in the pathophysiology of ischemic stroke [1]–[4], a leading cause of death worldwide. Today, the only approved treatment for stroke is thrombolysis with the recombinant tissue plasminogen activator (rt-PA) that improves outcomes in acute ischemic stroke patients by restoring cerebral blood flow. Nevertheless, its use remains limited to less than 5% patients due to its narrow therapeutic window of 4.5 hours [5] and the related risk of hemorrhagic transformations [6]. Moreover, rt-PA induces recanalization in only half of the treated patients [7] and early arterial reocclusion also occurs after successful thrombolysis in about 20 to 30% of recanalized patients [8]–[11]. Another major health concern in survival patients is the high risk of recurrent strokes within the following few weeks after the first event [12]. In addition to lifestyle changes and to the control of risk factors (e.g. hypertension, diabetes, dyslipidemia), current guidelines recommend antiplatelet agents (mostly aspirin and clopidogrel) as the fundamental strategy of secondary stroke prevention in patients with noncardioembolic disease [13]. However the modest benefit of these agents and the potential risk of bleedings point out the need for novel strategies [14]–[16].

Almost 10 years ago, Alexy and collaborators [17] demonstrated *in vitro* that three poly(ADP-ribose)polymerase (PARP) inhibitors (4-hydroxyquinazoline; 2-mercapto-4(3H)-quinazolinone; HO-3089) were able to reduce aggregation induced by adenosine diphosphate (ADP). PARP is an ubiquitous nuclear enzyme catalyzing the synthesis of poly(ADP-ribose) from nicotinamide adenine dinucleotide (NAD) and physiologically involved in DNA repair. As platelets are small anucleate cells, they theoretically cannot contain this enzyme. To our knowledge, there is no data reporting PARP presence in platelets, but we confirmed its absence by measuring the protein expression and enzyme activity in human platelets (data not shown). Therefore, the potential antiplatelet effect of PARP inhibitors would be PARP-independent as suggested in Alexy’s study [17]. Indeed, the authors attributed this effect to a potential competition between these inhibitors and ADP to bind to their platelet receptors, which might be due to a molecular structure resembling that of the adenine moiety of NAD and common with ADP. Such an inhibition of ADP-induced aggregation was not observed by Tóth and collaborators with INO-1001, another potent PARP inhibitor with a different structure [18]. Consequently, these data suggest that certain PARP inhibitors might exert antiplatelet effect and thus might prevent reocclusion after thrombolysis in ischemic stroke patients and/or be useful for secondary stroke prevention.

In pathophysiological conditions, such as stroke, the overactivation of PARP exerts deleterious effects, as demonstrated in several experimental models of cerebral ischemia [19], [20]. In rodent models of cerebral ischemia, we and others have shown that PJ34 (N-(6-oxo-5,6-dihydro-phenanthridin-2-yl)-N,N-dimethylacetamide), a potent PARP inhibitor (IC50 = 17 nM), reduces infarct volume, blood-brain barrier permeability, brain edema, spontaneous and rt-PA-induced hemorrhagic transformations, inflammatory response, motor deficit, and enhances long-term neuronal survival and neurogenesis [21]–[28].

**Figure 1:**
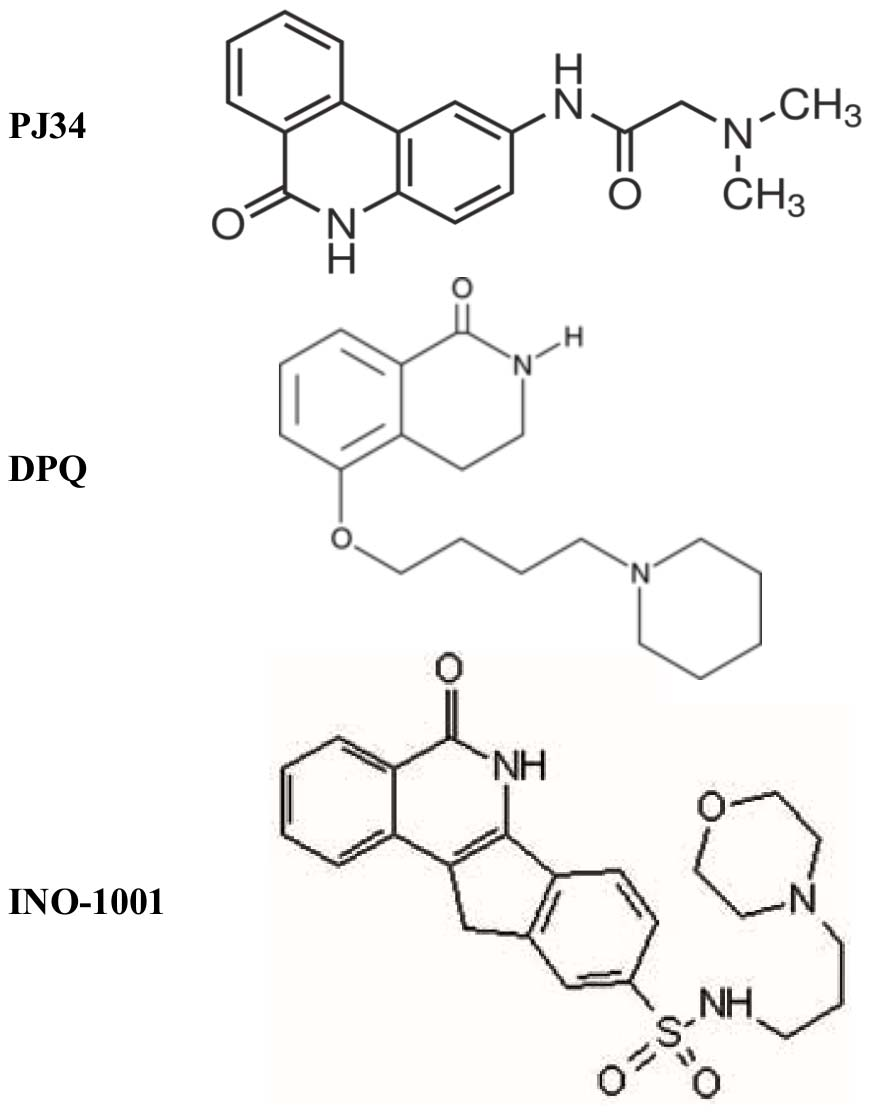
Chemical structure of the three PARP inhibitors: PJ34, DPQ and INO-1001.

In that context, the aim of our study was to evaluate *in vitro* on human blood whether PJ34 exerts antiplatelet effect and the potential mechanism involved. Such an effect, in addition to the protective effects mentioned above, would reinforce the interest of PJ34 in stroke treatment. The effect of two other PARP inhibitors, that have also demonstrated beneficial effects in experimental models of cerebral ischemia [29]–[31], but with different chemical structures, was also studied (Figure 1): a dihydroisoquinolinone (3,4-dihydro-5-[4-(1-piperidinyl)butoxy]-1(2H)-isoquinolinone or DPQ; IC50∼40 nM) and an isoindolinone derivative (INO-1001; IC50<15 nM). To our knowledge, this is the first work to report that PJ34 inhibits ADP-induced platelet aggregation in human platelet-rich plasma (PRP), probably acting *via* a P2Y_12_ pathway inhibition.

## Materials and Methods

### Chemicals and reagents

ADP was obtained from Roche (Boulogne-Billancourt, France) and PAR1ap (activating peptide of protease-activated receptor 1), used as a thrombin receptor activator, from Bachem (Bubendorf, Switzerland). Collagen was purchased from Nycomed (kit SKF Solution, Austria Takeda Pharmaceuticals International, Zurich, Switzerland).

PJ34 was purchased from Sigma-Aldrich (Saint Quentin Fallavier, France), DPQ from Santa Cruz Biotechnology (CliniSciences, Nanterre, France) and INO-1001 from Seleck Chemicals (Euromedex, Souffelweyersheim, France).

All reagents were dissolved and diluted in distilled water, except DPQ that was dissolved in 100% DMSO and diluted in distilled water.

### Human platelet aggregation studies

Venous blood from informed healthy donors was obtained from the French blood bank institute (EFS) according to the agreement between Paris Descartes University and EFS (C CPSL UNT No. 12/EFS/064).

Whole blood samples were collected from 35 volunteers into Vacutainer tubes containing 3.8% trisodium citrate (BD Vacutainer, Becton Dickinson, Le Pont de Claix, France) and centrifuged at 133 g for 20 minutes at 25°C (OrtoAlresa Digtor 21 R, Madrid, Spain) to obtain PRP. After carefully removing PRP, the pellet was centrifuged at 2630 g for 20 minutes at 25°C to obtain platelet-poor plasma (PPP). The final platelet count in PRP samples was adjusted to 2.5×10^8^ platelets/ml with autologous PPP.

Platelet aggregation was quantified in PRP by optical aggregometry using a PAP-8E Aggregometer (Bio/Data Corporation, Horsham, PA, USA) with a constant stirring rate of 1000 rpm at 37°C.

Ten microliters of PARP inhibitors (PJ34, DPQ or INO-1001) or vehicle (distilled water or 0.3% DMSO) were added to 230 µl of PRP in glass cuvettes. Samples were pre-incubated at 37°C for 10 minutes. After baseline adjustment, 10 µl of platelet agonist (ADP, collagen or PAR1ap) were added and aggregation was recorded for 8 minutes. A glass cuvette containing 20 µl of vehicle (distilled water or 0.3% DMSO) and 230 µl of PPP served as a control of “100% light transmission”.

Agonist concentrations were adjusted for each donor to induce a maximum aggregation of about 65–70%: collagen (1−1.5 or 2 µg/ml) and PAR1ap (1−1.5 or 2 µM). Moreover, ADP concentration has to be determined for each donor to induce a biphasic aggregation curve (1.5−2–2.5−3–4 or 5 µM).

PJ34 was used at 1, 10, 25, 50 or 100 µM and the other PARP inhibitors (DPQ and INO-1001) at 50 µM.

References ( = platelet agonists alone) were normalized to 100%. The maximal aggregation (%) measured in the presence of PARP inhibitor was compared to that of the corresponding reference, except for ADP-induced aggregation where maximal aggregation was distinguished for each phase.

To evaluate the antiaggregant effect of PJ34 on platelets stimulated with increasing ADP concentrations (1.5 to 10 µM), PRP was pre-incubated with fixed concentrations of PJ34 (50, 100 or 500 µM). In this experiment, since the ADP concentration used did not always induce a biphasic aggregation curve, the maximum aggregation (%) in the presence of PJ34 was compared to the corresponding reference.

The concentrations of PARP inhibitors, platelet agonist or DMSO mentioned above correspond to the final concentration in 250 µl.

### VASP phosphorylation

Vasodilator stimulated phosphoprotein (VASP) phosphorylation was measured using a modified commercial kit (PLT VASP/P2Y_12_ assay, Biocytex, Marseille, France) on a FACS Calibur flow cytometer (Becton Dickinson, Franklin Lakes, USA). VASP is an intracellular platelet protein which phosphorylation is regulated by cyclic adenosine monophosphate (cAMP) and activation of a protein kinase pathway. Prostaglandin E1 (PGE1) activates this pathway resulting in VASP phosphorylation, whereas ADP inhibits this cascade through P2Y_12_ receptors and thus reduces VASP phosphorylation.

Briefly, citrated whole blood from 4 healthy volunteers was pre-incubated for 10 minutes with PJ34 (final concentrations: 100, 250, 500 or 1000 µM) or its vehicle (distilled water). The results were expressed as the platelet reactivity index (PRI).

PRI is calculated using corrected mean fluorescence intensities (MFI) in the presence of (1) PGE1 alone (MFI PGE1) or (2) PGE1+ ADP associated with PJ34 or its vehicle (MFI (PGE1+ADP±PJ34)), as follows:

PRI = [(MFI PGE1– MFI (PGE1+ADP±PJ34))/MFI PGE1 ] × 100.

One has to know that a low PRI is associated with a high VASP phosphorylation state, and therefore to an inhibition of ADP-induced platelet response.

### Statistical analysis

Data are expressed as mean ± SD.

For PJ34, samples were compared with the vehicle group ( = platelet agonist alone) using a one-way analysis of variance (ANOVA) followed by Dunnett's test.

For DPQ and INO-1001, comparisons with the vehicle group were evaluated by one-way ANOVA followed by Student t test.

Pearson correlation test was used to evaluate the relationship between PJ34 concentrations and ADP-induced platelet aggregation, and between PJ34 concentrations and PRI.

Statistical significance was assigned at P<0.05, after Bonferroni corrections for multiple comparisons when necessary.

All tests were performed with GraphPad Prism 5 (GraphPad Software, Inc., La Jolla, USA).

## Results

### Effect of PJ34 on ADP-induced platelet aggregation

**Figure 2:**
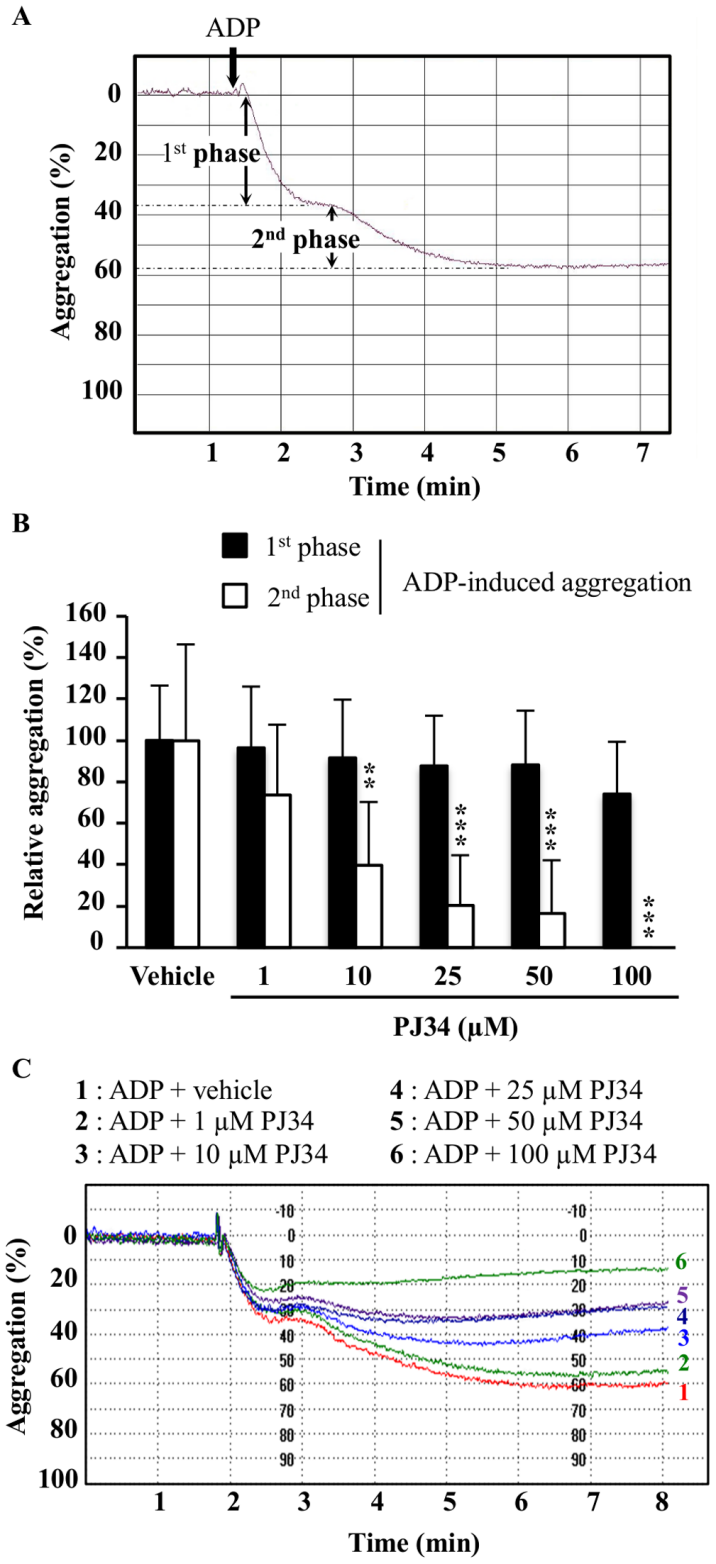
Effect of PJ34 on both phases of ADP-induced platelet aggregation. (A) ADP concentration (1.5 to 5 µM) was determined for each donor to produce a biphasic aggregation curve. (B) Human PRP samples were pre-incubated with PJ34 (1–100 µM) or its vehicle and then stimulated with ADP. Results are expressed relatively to ADP alone ( = vehicle group; normalized to 100%) and presented as the mean ± SD (n = 6–7). **P<0.01 and ***P<0.001 *versus* the corresponding vehicle group (one-way ANOVA followed by Dunnett's test). (C) Representative curves showing the effect of PJ34 or its vehicle on platelet aggregation induced by 2 µM ADP.

The antiplatelet effect of PJ34 was first tested on ADP-induced PRP aggregation. ADP concentration has to be determined for each donor in order to induce a biphasic aggregation curve resulting from the activation of ADP receptors, P2Y_1_ and P2Y_12_, successively. Before normalization, basal maximal aggregation was about 64±9.8% with the first phase at 40±10.6% and the second one at 24±11.9%, as shown on the representative curve (Figure 2A).

PJ34 had no effect on the first phase of ADP-induced platelet aggregation, but inhibited the second one in a concentration-dependent manner (r = −0.617; P<0.001) by 60% at 10 µM (P<0.01), by 80% at 25 µM and 50 µM (P<0.001) and totally at 100 µM (P<0.001; Figures 2B and C).

**Figure 3:**
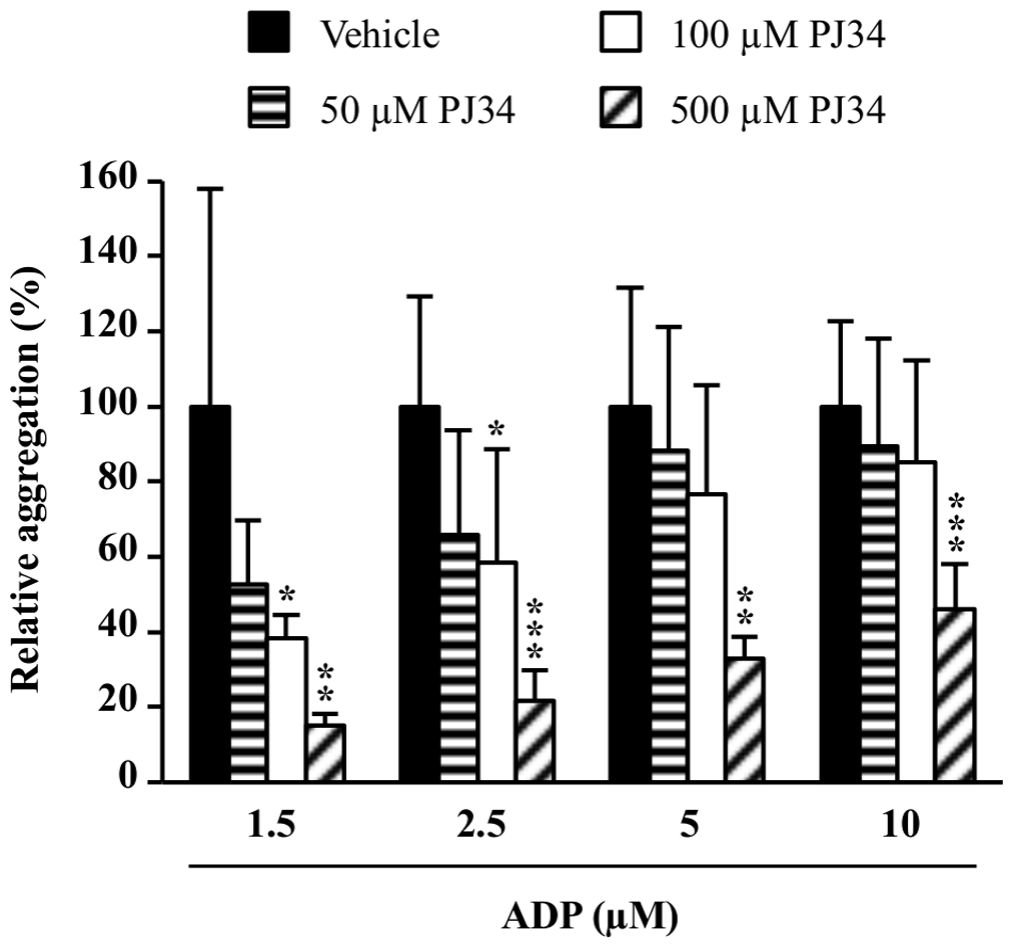
Effect of incremental concentrations of ADP on platelet aggregation in the presence of PJ34. Human PRP samples were pre-incubated with PJ34 or its vehicle and then stimulated with ADP. Results are expressed relatively to ADP alone ( = vehicle group; normalized to 100%) and presented as the mean ± SD (n = 4–7). *P<0.05, **P<0.01 and ***P<0.001 *versus* the corresponding vehicle group (one-way ANOVA followed by Dunnett's test).

Considering the concentration-dependent effect of PJ34 on the second phase of ADP-induced platelet aggregation, we then evaluated the potential competition between ADP and PJ34 using incremental concentrations of ADP. Figure 3 shows that the more ADP concentrations increased (up to 10 µM) the less PJ34 had an inhibitory effect. For instance, 100 µM of PJ34 reduced by 60% the platelet aggregation induced by 1.5 µM of ADP (P<0.05), by 40% the 2.5 µM ADP-induced platelet aggregation (P<0.05), by 25% the platelet aggregation induced by 5 µM and by 15% the 10 µM ADP-induced platelet aggregation.

### Effect of PJ34 on VASP phosphorylation

Because the second aggregation phase induced by ADP is mainly dependent on the engagement of P2Y_12_ receptor, leading to the inhibition of VASP phosphorylation, we used flow cytometry quantification of phosphorylated VASP (P-VASP) to investigate the interaction of PJ34 with P2Y_12_ signaling. This method is one of the most valuable to evaluate the efficacy of P2Y_12_ antagonists, such as clopidogrel [32]. In the test, a low PRI is associated with a high VASP phosphorylation state.

**Figure 4:**
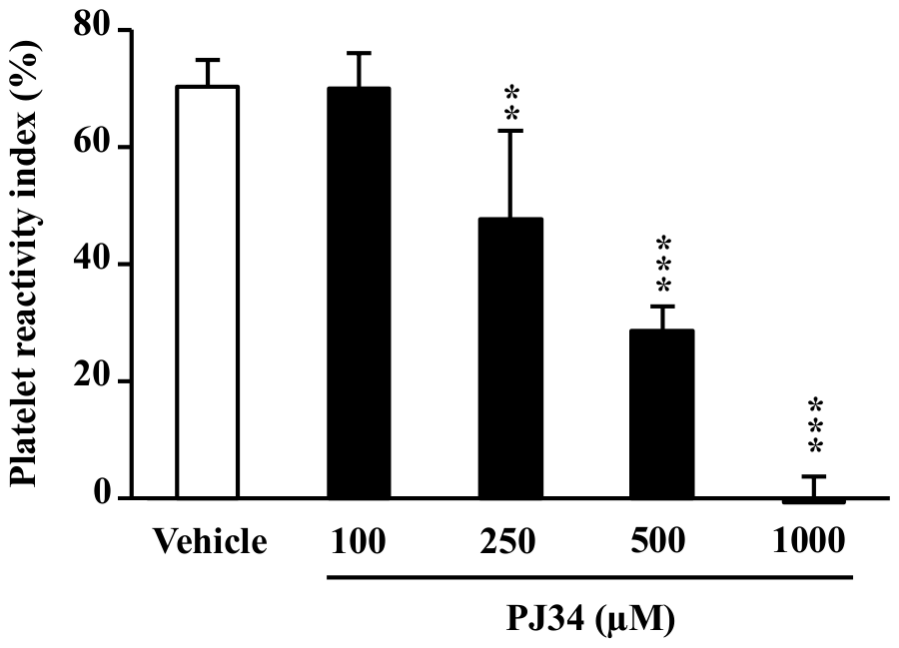
Effect of PJ34 on VASP phosphorylation. Human whole blood was pre-incubated with PJ34 or its vehicle and then stimulated with ADP. A low platelet reactivity index (PRI) is associated with a high VASP phosphorylation state and thus reflects P2Y_12_ inhibition. Results are expressed relatively to ADP alone ( = vehicle group) and presented as the mean ± SD (n = 3–4). **P<0.01 and ***P<0.001 *versus* vehicle (one-way ANOVA followed by Dunnett's test).

The PRI of ADP alone was 70±4.6%. PJ34 reduced the PRI in a concentration-dependent manner (r = −0.951; P<0.001) by 30% at 250 µM (P<0.01), 60% at 500 µM (P<0.001) and totally at 1000 µM (P<0.001; Figure 4).

### Effect of PJ34 on collagen- and PAR1ap-induced platelet aggregation

**Figure 5:**
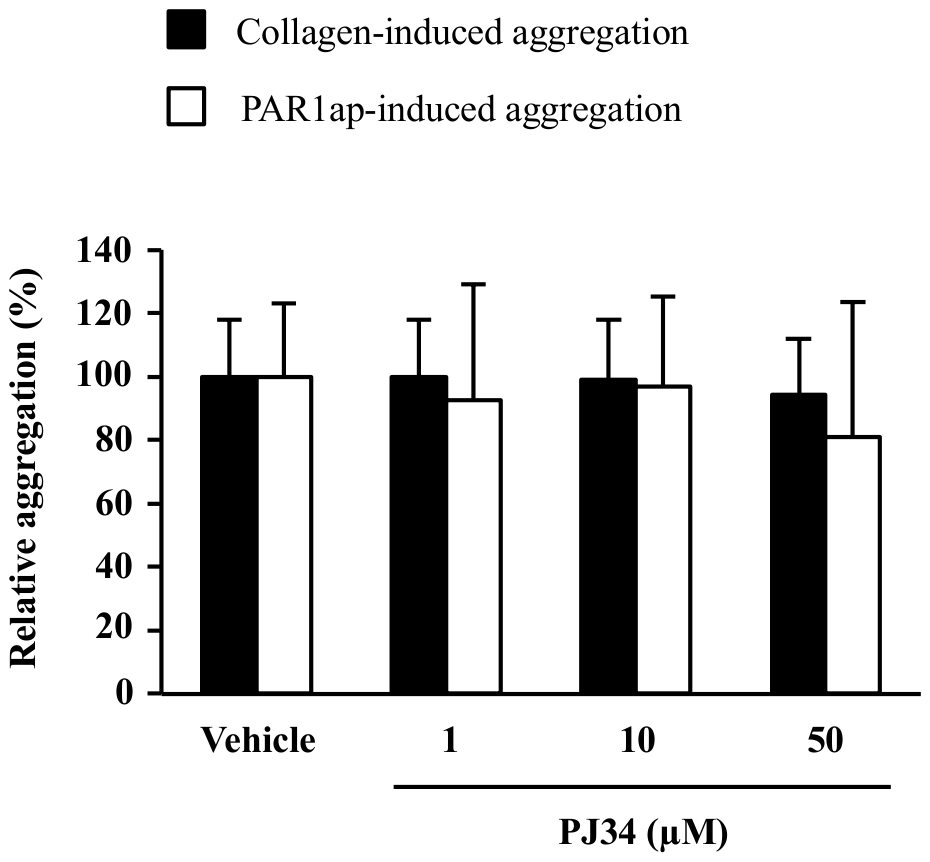
Effect of PJ34 on collagen- and PAR1ap-induced platelet aggregation. Human PRP samples were pre-incubated with PJ34 or its vehicle and then stimulated with either collagen (1 to 2 µg/ml) or PAR1ap (1 to 2 µM). Results are expressed relatively to platelet agonist alone ( = vehicle group; normalized to 100%) and presented as the mean ± SD (n = 7–8).

In order to determine the specificity of PJ34 towards ADP-induced platelet aggregation, we next investigated the inhibitory effect of PJ34 on two other platelet agonists (collagen and PAR1ap). Since it is well known that secreted ADP constitutes a second feedback amplification signal in the setting of platelet aggregation, we used agonist concentrations inducing a strong activation less dependent, or independent, on secreted ADP. We thus checked that the aggregation induced by these concentrations was not inhibited (or very slightly) by the selective P2Y_12_ receptor antagonist 2MesAMP (2-methylthioadenosine 5′-monophosphate triethylammonium salt; data not shown) [33]. In these conditions, whatever the PJ34 concentration, no inhibitory effect was observed on either collagen- or PAR1ap-induced platelet aggregation (Figure 5).

### Effect of two PARP inhibitors with different chemical structures on ADP-, collagen- and PAR1ap-induced platelet aggregation

**Figure 6:**
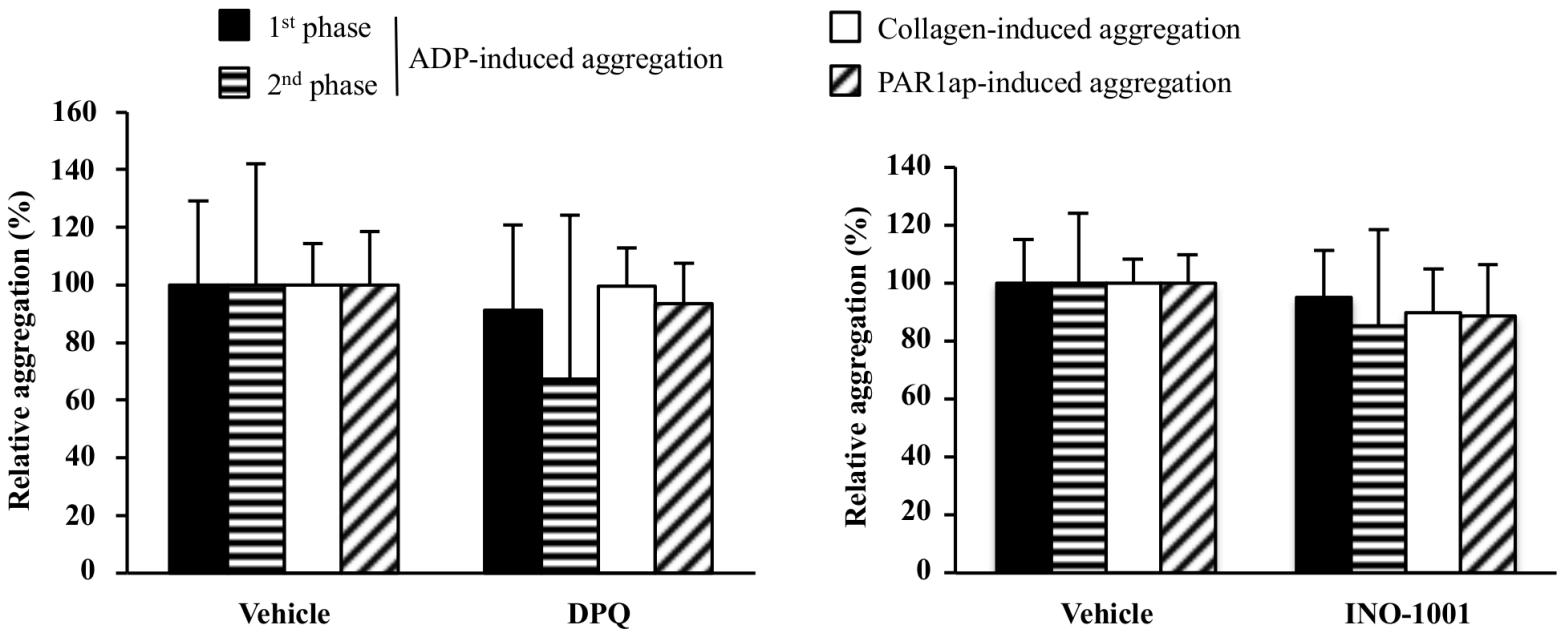
Effect of DPQ and INO-1001 on ADP-, collagen- or PAR1ap-induced platelet aggregation. Human PRP samples were pre-incubated with PARP inhibitor (50 µM) or its vehicle and then stimulated with ADP (1.5 to 5 µM; to produce a biphasic aggregation curve), collagen (1 to 2 µg/ml) or PAR1ap (1 to 2 µM). Results are expressed relatively to platelet agonist alone ( = vehicle group; normalized to 100%) and presented as the mean ± SD (n = 5–6).

To evaluate whether the inhibitory effect on ADP-induced platelet aggregation was specific to PJ34, we evaluated the effect of two structural different PARP inhibitors, DPQ and INO-1001, at 50 µM. No significant effect of these inhibitors could be observed, although the second phase of ADP-induced platelet aggregation tended to be reduced by DPQ (Figure 6). DPQ and INO-1001 were devoid of effect on collagen- and PAR1ap-induced platelet aggregation in our experimental conditions (as explained above).

## Discussion

Beside their physiological role in maintaining hemostasis, platelets are also involved in the formation of pathogenic thrombi. Platelet activation is triggered by a variety of agonists, such as sub-endothelial collagen, thromboxane A2 and ADP released from activated platelets or thrombin generated by the coagulation cascade [34]. These agonists act on different platelet receptors, but they all trigger signaling pathways leading to intracellular calcium concentration increase [35]. ADP, which is a weak agonist compared to collagen or thrombin, is however considered as the main amplifier of initial aggregation.

In this study, we showed that PJ34 inhibits ADP-induced platelet aggregation. This antiplatelet effect seems specific to ADP receptors since PJ34 does not inhibit collagen- or PAR1ap-induced platelet aggregation; both these agonists were used at concentrations inducing a strong activation less dependent, or independent, on secreted ADP. Our results are in accordance with those of Alexy and collaborators [17] who investigated the effects of three other PARP inhibitors (4-hydroxyquinazoline; 2-mercapto-4(3H)-quinazolinone; HO-3089) and found that they reduced ADP-, but not epinephrine- or collagen-induced platelet aggregation, presumably due to a competition between these PARP inhibitors and ADP for binding to its receptors.

To go further in this hypothetical mechanism, we examined which ADP receptor(s), i.e. P2Y_1_ and/or P2Y_12_, might be implicated. Indeed, the mechanism of ADP-induced platelet aggregation is complex, since the co-activation of both G protein-coupled receptors P2Y_1_ and P2Y_12_ is necessary to produce a complete aggregation response [36], [37]. The Gq coupled-P2Y_1_ receptor is essential in the initiation of ADP-induced platelet activation; it mediates calcium mobilization from internal stores, which results in platelet shape change, and weak and transient aggregation. The Gi coupled-P2Y_12_ receptor activation, *via* lowering cAMP levels induces the amplification of platelet aggregation and secretion; this receptor plays a pivotal role in sustaining platelet aggregation. For that purpose, ADP concentrations were determined to produce a biphasic aggregation curve. In these conditions, PJ34 (10 to 100 µM) only inhibited the second phase of ADP-induced platelet aggregation without modifying the first one, suggesting that its antiaggregant effect is specifically linked to P2Y_12_.

Moreover, we observed that incremental ADP concentrations (1.5 to 10 µM) reduced the inhibitory effect of PJ34 (50, 100 and 500 µM), and that the antiaggregant effect of 100 µM PJ34 even disappears when applying ADP at 10 µM, suggesting a competitive effect of PJ34 with ADP for P2Y_12_. In order to test this hypothesis, we assessed by flow cytometry the phosphorylation state of the intracellular platelet protein VASP. This assay is commonly used in clinical practice to evaluate the efficiency of P2Y_12_ antagonism in patients treated with clopidogrel [32]. Therefore, any specific P2Y_12_ antagonist impedes VASP dephosphorylation, and consequently, promotes the phosphorylated form. This assay had to be performed with higher concentrations of PJ34 (100 to 1000 µM) than in aggregation studies. On one hand, these experimental conditions were partly attributed to the fact that whole blood has to be used with the commercial kit (PLT VASP/P2Y_12_ assay) that does not work on PRP. It is important to note that, in both blood and plasma, ADP can be converted to adenosine, an inhibitor of platelet aggregation. However, adenosine is readily removed from blood through uptake into red blood cells, which makes it unavailable to inhibit platelet aggregation. Therefore, adenosine can exert more efficiently its inhibitor effect on platelet aggregation in PRP where red blood cells are absent [38]. This could explain that lower PJ34 concentrations are necessary to inhibit ADP-induced platelet aggregation in PRP. On the other hand, as the concentration of ADP provided in the kit is not specified, this concentration may be higher than those we used before. Whatever the reason, our results show that PJ34 increased the amount of P-VASP in a concentration-dependent manner, suggesting that PJ34 may act like a P2Y_12_ antagonist.

Since our original hypothesis relied on a “PARP-independent” mechanism, we then examined whether two other PARP inhibitors with different chemical structure exert similar antiplatelet effect. We opted for DPQ and INO-1001 that also demonstrated beneficial effects in experimental models of cerebral ischemia [29]–[31]. The concentration of these PARP inhibitors (50 µM) was chosen based on the PJ34 concentration inducing an important antiaggregant effect, without referring to their IC50 for PARP accordingly to our hypothesis.

Neither DPQ nor INO-1001 modified platelet aggregation whatever the agonist used, although we noticed that the second phase of ADP-induced platelet aggregation tended to be inhibited by DPQ. However, due to the important variability between donors, this inhibition was not significant. Results with INO-1001 are consistent with previous data showing no effect of INO-1001 at 10 or 100 µM [18]. The authors suggested that this lack of antiplatelet effect might simply be explained by its structure, but they also suspected the concentrations used. Indeed, in Toth’s study, INO-1001 concentrations are consistent with those preventing PARP activation in a cardiomyocyte oxidative challenge model or even with plasma concentrations measured in *in vivo* models, whereas Alexy and collaborators [17] showed that inhibition of ADP-induced platelet aggregation with their PARP inhibitors occurred at higher concentrations than those inhibiting PARP activity in similar cell culture.

In our study, PJ34 significantly inhibited the second phase of ADP-induced platelet aggregation by 60% at 10 µM, with a tendency at 1 µM (25%). Although these concentrations are higher than the IC50 for PARP inhibition (17 nM), they are similar to those used by Tóth and collaborators with the equipotent PARP inhibitor, INO-1001 (IC50<15 nM). Moreover, PJ34 concentrations are consistent with those commonly used in *in vitro* studies. So, for example, pre-treatment with 30–1000 nM PJ34 significantly protected neurons from cell death induced by oxygen and glucose deprivation in a dose-dependent manner [21]. PJ34 (10 or 100 µM) also protected cardiomyoblast cells against H_2_O_2_-induced injury [39]. In human umbilical vein endothelial cell culture, Mathews and Berk [40] showed that PJ34 at 10 µM reduced H_2_O_2_-induced cell death.

Other authors have already pointed out that PJ34 possessed PARP-independent properties [41]–[43]. Our results hereby highlight another specific facet of this PARP inhibitor that reinforces its superiority *versus* the others.

In conclusion, our study is the first one demonstrating that the PARP inhibitor PJ34 inhibits ADP-induced platelet aggregation *via* a potential competitive P2Y_12_ antagonism. Moreover, since it is well established that pharmacological blockade of P2Y_12_, either with thienopyridines such as clopidogrel or prasugrel or with the direct inhibitor ticagrelor, is a powerful antiplatelet strategy, this antiplatelet effect strengthens the interest of PJ34 in stroke treatment. Indeed, our results suggest that, in addition to its already demonstrated neuro- and vasoprotective effects in cerebral ischemia, PJ34 might also improve spontaneous or rt-PA-induced recanalization and/or prevent from early reocclusion.
